# Physicians Acting the Dentist

**Published:** 1848-07

**Authors:** A. Berry

**Affiliations:** Cincinnati


### PHYSICIANS ACTING THE DENTIST.

BY A. BERRY, D. D. S.—CINCINNATI.

"In many parts of the southern states, physicians are much engaged, during the sickly season, in the fall and during the rest of the year, have almost complete leisure. How profitable, if they were to qualify themselves in the way we have here indicated, might they employ their leisure time, both to themselves and to the community in which they live. This is a subject of much importance, and should be taken into consideration by all students of medicine who design practicing in the country."—*Treatise on the Diseases of the Teeth, by R. A. Arthur, Phila. page* 185.

---

It might be thought that dentists at the east had seen malpractice enough in their profession by physicians and medical students, to prevent them from advising individual men to attempt to combine the practice of medicine and dentistry.

A southern physician is commonly a professor of things in general. He has usually considerable practice in the winter and spring, as well as during the sickly season. Besides his professional business, he attends to the affairs of his family, looks after his negroes, talks politics with his neighbors, and acts on committees and make speeches at political meetings. During the little leisure he may have in the winter, he must spend much time in collecting his bills—often more difficult to collect than to make. Consequently he has no time to devote to the practrce of dentistry.

Few physicians would spend sufficient time in the study of dentistry, unless they intend to confine their attention solely to it, to become proficient in it. No art requires more skill, the result of thorough instruction, than that of filling teeth. No man, however successful and skillful he may be as a physician or surgeon, should attempt to fill or insert teeth short of from one to two years of close study of the principles and practice of dentistry. In nothing except medicine is a little learning so dangerous a thing as in dentistry; Although a knowledge of anatomy, physiology, pathology, therapeutics and chemistry is essential for the dentist, it does not by any means qualify him for the practice of his profession. I have known several physicians in a county, in the south, who made considerable pretensions to dental acquirements, but whose filling and inserting teeth were, in my humble opinion, worse than worthless.

The practice of either medicine or dentistry, is of itself amply sufficient to occupy the attention and talents of any man. If he would excel in either, he must confine his studies and labors to it exclusive of the other. The old adage, "too many irons in the fire," applies to physicians engaged in other business besides their professional duties with more force than to any other men. Our profession suffers from quackery sufficiently without furtherance in this respect by physicians.

